# Posttreatment squamous cell carcinoma antigen: A prognostic marker for patients with advanced cervical cancer treated with bevacizumab–paclitaxel–cisplatin therapy

**DOI:** 10.1097/MD.0000000000044673

**Published:** 2025-09-19

**Authors:** Haa-Na Song, In Bong Ha, Bae Kwon Jeong, Jae Min Cho, Bong Hoi Choi, Jeong Hee Lee, Jeong Kyu Shin

**Affiliations:** a Department of Internal Medicine, Division of Hematology-Oncology, Gyeongsang National University of Medicine and Gyeongsang National University Hospital, Jinju, Republic of Korea; b Institute of Health Science, Gyeongsang National University, Jinju, Republic of Korea; c Biomedical Research Institute, Gyeongsang National University Hospital, Jinju, Republic of Korea; d Department of Radiation Oncology, Gyeongsang National University of Medicine and Gyeongsang National University Hospital, Jinju, Republic of Korea; e Department of Radiology, Gyeongsang National University of Medicine and Gyeongsang National University Hospital, Jinju, Republic of Korea; f Department of Nuclear Medicine, Gyeongsang National University Hospital, Gyeongsang National University School of Medicine, Jinju, Republic of Korea; g Department of Pathology, Gyeongsang National University of Medicine and Gyeongsang National University Hospital, Jinju, Republic of Korea; h Department of Obstetrics and Gynecology, Gyeongsang National University of Medicine and Gyeongsang National University Hospital, Jinju, Republic of Korea.

**Keywords:** bevacizumab, cervical cancer, cisplatin, paclitaxel, squamous cell carcinoma antigen

## Abstract

Cervical cancer is a leading cause of cancer-related mortality in women, with poor prognosis when diagnosed at advanced stages. Bevacizumab, an anti-vascular endothelial growth factor agent, has shown promising results in the treatment of metastatic cervical cancer. The combination of bevacizumab with paclitaxel and cisplatin (TP) as first-line chemotherapy is associated with improved survival rates in patients with metastatic cervical cancer. In this study, we aimed to evaluate the effectiveness and safety of first-line chemotherapy with bevacizumab and TP in patients with advanced cervical cancer in South Korea and investigate the clinical value of posttreatment plasma levels of squamous cell carcinoma antigen (SCC Ag) as an independent prognostic factor for survival. This retrospective study included 33 patients with metastatic cervical cancer who received bevacizumab and TP as first-line chemotherapy between January 2016 and December 2022 at the Gyeongsang National University Hospital. First-line therapy with bevacizumab and TP significantly improved progression-free and overall survival (OS) [26.3 (95% confidence interval (CI), 18.3–34.3) and 39.9 (95% CI, 23.8–56) months, respectively]. Patients who achieved normalization of posttreatment SCC Ag levels had significantly longer OS compared to those who did not [69.1 (95% CI, 17.6–120.6) vs 18.4 (95% CI, 10.9–25.9) months, respectively (hazard ratio 12.4; *P < *.001)]. The treatment regimen was well-tolerated, with most patients reporting manageable side effects. First-line chemotherapy with bevacizumab and TP demonstrated efficacy and tolerability in Korean patients with metastatic cervical cancer. Additionally, normalization of posttreatment SCC antigen levels serves as a potential prognostic marker for patients undergoing this type of chemotherapy.

## 1. Introduction

Cervical cancer is the fourth most common cancer and remains the leading cause of cancer-related mortality among women worldwide. In 2020, approximately 604,000 new cervical cancer cases were diagnosed globally, with an estimated 342,000 deaths.^[1,2]^ In the Republic of Korea, 2998 new cases were reported during the same year, rendering cervical cancer the tenth most common cancer among women.^[3]^ The increased detection of early-stage cervical cancer has been largely attributed to the widespread adoption of pap smear screening and human papillomavirus (HPV) vaccination programs.^[4]^ Despite these advancements, the prognosis for patients diagnosed with late-stage cervical cancer remains poor, with a 5-year survival rate of only 20%.

To address this unmet clinical need, novel chemotherapeutic agents have been developed to improve the outcomes of advanced cervical cancer. Bevacizumab, a humanized monoclonal immunoglobulin G (IgG) antibody that targets the vascular endothelial growth factor receptor (VEGF), has emerged as a promising anti-angiogenic agent.^[5]^ The pivotal GOG-240 trial, published in 2014, demonstrated that the addition of bevacizumab to standard first-line chemotherapy significantly improved overall survival (OS) in patients with advanced cervical cancer.^[6–11]^ Specifically, the combination of bevacizumab with 2 distinct chemotherapy doublets significantly improved OS when compared to chemotherapy alone [17.0 months vs 13.3 months; hazard ratio (HR): 0.71, 98% confidence interval (CI): 0.54–0.95; *P = *.004].^[6,7]^ However, the generalizability of these findings is limited by the demographic composition of the study population, which primarily included patients from Western countries. This raises concerns regarding the applicability of these results in real-world clinical settings in Asia, including Korea.

While the combination of novel chemotherapeutic agents demonstrates improved survival, current prognostic indicators have certain limitations in the real world. Additional biomarkers are required to predict patient response to treatment. Serum squamous cell antigen (SCC Ag) is commonly used as a tumor marker for early diagnosis and monitoring cervical cancer relapse. Previous studies have demonstrated that high concentrations of pretreatment SCC Ag are significantly associated with poor prognosis in patients with early-stage cervical cancer.^[12,13]^ However, there is a lack of evidence regarding SCC Ag monitoring and whether it may serve as a predictive biomarker of treatment response in patients with advanced cervical cancer who receive bevacizumab containing chemotherapy.

Therefore, in this study, we aimed to evaluate the usefulness of the SCC Ag as a marker for predicting treatment response in patients with advanced cervical cancer. Moreover, we aimed to demonstrate the real-world efficacy and safety of a combination therapy of paclitaxel and cisplatin (TP) plus bevacizumab in patients with advanced cervical cancer in the Republic of Korea.

## 2. Materials and methods

### 2.1. Participants

This retrospective study included 33 patients with metastatic cervical cancer who received bevacizumab as part of the first-line chemotherapy between January 2016 and December 2022. The inclusion criteria were as follows: age ≥ 18 years, histologically confirmed invasive cervical cancer, radiologically confirmed distant metastasis at bevacizumab treatment initiation, and receipt of chemotherapy with bevacizumab. Patients who received prior chemotherapy or underwent targeted therapy for metastatic cervical cancer, were diagnosed with other malignancies within 5 years, had inappropriate laboratory findings, or had severe comorbidities contraindicating standard chemotherapy were excluded from the study.

All patients received a chemotherapy regimen comprising an intravenous infusion of bevacizumab (15 mg/kg over 90 minutes), paclitaxel (175 mg/m^2^ over 3 hours), and cisplatin (60 mg/m^2^ over 1 hour), administered every 3 weeks. Prior to the first chemotherapy cycle, the patients underwent comprehensive clinical evaluation, including medical history taking, complete blood count and serum chemistry, chest radiography, and computed tomography of the involved sites. Therapy was continued until disease progression according to the Response Criteria in Solid Tumors (RECIST),^[12]^ the occurrence of unacceptable toxicity, deterioration of clinical condition, or patient withdrawal of consent. For each subsequent cycle, the patients were required to meet the following criteria: absolute neutrophil count ≥ 1500 m^3^/dL, platelet count ≥ 75,000 m^3^/dL, serum creatinine ≤ 1.5 mg/dL, and non-hematologic adverse events ≤ grade 2. The dose adjustments were based on the most severe toxicity observed during the preceding cycle. Treatment was discontinued in cases of severe hypersensitivity reactions or unacceptable toxicity.

### 2.2. Clinical data collection and definitions

Clinical and laboratory data were retrospectively collected through a review of the electronic medical records. Patients with missing pathological or key clinical data were excluded from analysis. The following baseline parameters were obtained at the time of chemotherapy initiation: age, body mass index, pre- and posttreatment SCC Ag, Eastern Cooperative Oncology Group performance status, presence of comorbidities such as diabetes and dyslipidemia, and interval between initial diagnosis and initiation of bevacizumab therapy. The normal range of Serum SCC Ag was 0.0 ~ 1.5 ng/mL, serum SCC Ag level was checked at the time of diagnosis of cervical cancer and within a month of completing chemotherapy, which means posttreatment SCC Ag.^[13,14]^ Treatment responses were evaluated every 3 cycles using chest and abdominopelvic CT in accordance with the RECIST criteria. Adverse events were documented and graded according to National Cancer Institute criteria. Structured medical record reviews were conducted to identify causes of treatment discontinuation and death. The primary study endpoint was progression-free survival (PFS). The secondary endpoints included OS, overall response rate, and treatment-related safety. The study protocol was reviewed and approved by the Institutional Review Board of the Gyeongsang National University Hospital (approval number: GNUH 2023-11-008-002). The requirement for informed consent was waived owing to the retrospective nature of the study.

### 2.3. Statistical analysis

Standard descriptive and analytical methods were used to summarize the demographic and baseline clinical characteristics. Serum SCC was defined as the interval from the initiation of bevacizumab treatment to the date of documented disease recurrence, while OS was defined as the interval from the initial diagnosis to the date of death. Survival outcomes were estimated using the Kaplan–Meier method. The effects of baseline clinical and laboratory variables on PFS and OS were analyzed using the Cox proportional hazards model. Laboratory parameters and age were assessed as continuous and categorical variables. Statistical significance was set at *P < *.05, with all *p*-values derived from 2-sided tests. Statistical analyses were performed using IBM SPSS Statistics for Windows version 22 (IBM Corp., Armonk).

## 3. Results

### 3.1. Patient characteristics

The baseline demographic characteristics of the 33 patients are summarized in Table 1. All eligible patients were female, with a median age of 56 years (range, 28–74 years). Approximately half of the enrolled patients (n = 16, 48.5%) had recurrent disease and 93.8% (n = 15) had undergone prior radiation therapy or concurrent chemoradiotherapy. The median follow-up period, defined as the time from diagnosis to confirmed survival or death, was 31.3 months. The median duration of bevacizumab therapy was 10 months, with a median of 7 bevacizumab treatment cycles (range: 2–33 months and 1–25.6 cycles, respectively).

**Table 1:** Baseline clinical characteristics of the participants (n = 33).

Characteristics	No. of patients	(%)
Age, years, median (range)	56 (28–74)	–
BMI (kg/m^2^, median, range)	24.2 (12.5–30)	–
Obesity, diagnosed based on BMI		
Yes	13	39.4
No	20	60.6
ECOG performance status		
0	8	24.2
1	20	60.6
2	5	15.2
Histology		
Squamous cell carcinoma	29	87.9
Adenocarcinoma	4	12.1
SCC Ag normalization during treatment		
Yes	25	75.8
No	8	24.2
Total cycles of bevacizumab treatment, median (range)	10 (2–33)	–
Duration of bevacizumab treatment, months, median (range)	7 (1–25.6)	–
Duration of follow-up, months, median (range)	31.3 (9.4–87.5)	–

BMI = body mass index, ECOG = Eastern Cooperative Oncology Group, SCC Ag = squamous cell carcinoma antigen.

### 3.2. Efficacy

**Figure 1.**
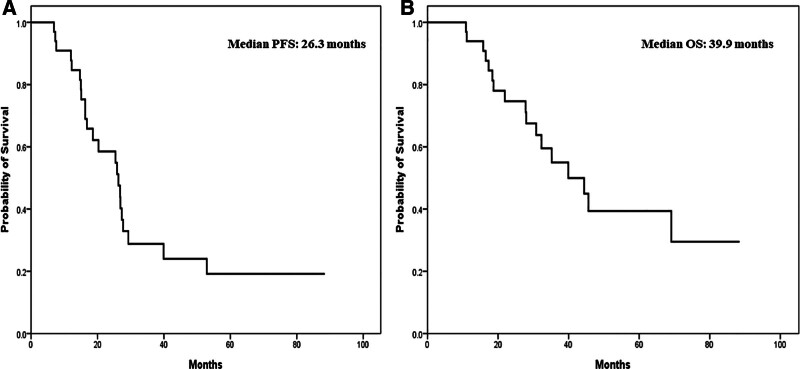
Survival analysis of the participants (n = 33). (A) The median PFS was 26.3 (95% CI, 18.3–34.3) mo; (B) median OS was 39.9 (95% CI, 23.8–56) mo. CI = confidence interval, OS = overall survival, PFS = progression-free survival.

During the observation period, 22 patients experienced disease relapse, and 17 succumbed to the disease. For the 33 participants included in the analysis, the median PFS and OS were 26.3 (95% CI, 18.3–34.3) and 39.9 (95% CI, 23.8–56) months, respectively. The Kaplan–Meier estimates for both PFS and OS are shown in Figure 1. Notably, most patients achieved either complete (n = 18, 54.5%) or partial (n = 13, 39.4%) response after receiving bevacizumab and TP.

**Figure 2.**
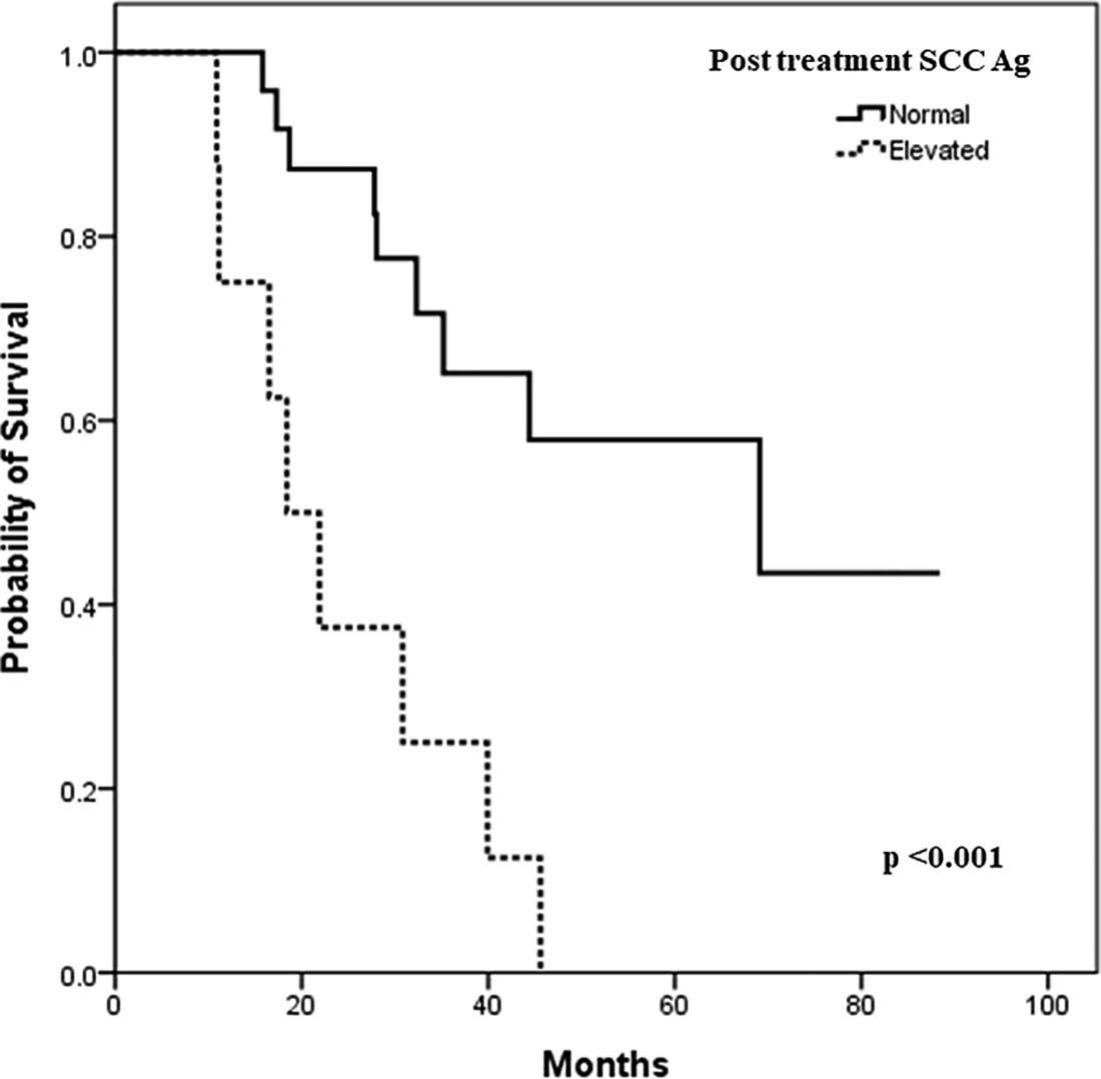
Overall survival stratified by the normalization of posttreatment SCC Ag. The median OS of patients who achieved normalization of posttreatment SCC Ag was significantly longer than that of patients with elevated posttreatment SCC Ag (69.1 mo vs 3.82 mo, respectively, *P < *.001). OS = overall survival, SCC Ag = squamous cell carcinoma antigen.

To evaluate the prognostic factors, we analyzed the HRs of various clinical and laboratory parameters associated with survival using the Cox regression model (Fig. 2). Patients who exhibited normalization of SCC Ag during treatment had significantly longer survival than those who did not normalize SCC Ag. The median OS for patients with normalized SCC Ag was 69.1 (95% CI, 17.6–120.6) months compared to 18.4 (95% CI, 10.9–25.9) months for those without normalization, with an HR of 12.4 (*P < *.001). No other clinical or laboratory parameters significantly affected PFS or OS in the Cox regression model, except for SCC Ag.

### 3.3. Safety

Chemotherapy was delayed in 2 patients (6.1%) due to intolerable peripheral neuropathy; however, none of the patients discontinued treatment solely due to chemotherapy-related toxicity. The most common reason for chemotherapy discontinuation was disease progression. Overall, the combination of bevacizumab and TP chemotherapy was generally well-tolerated, with azotemia and peripheral neuropathy being the most frequently observed toxicities (Table 2).

**Table 2:** Adverse events that are potentially associated with chemotherapy (n = 33).

Adverse events	All grades, N (%)	Grade 3 or 4, (%)
Anemia	3 (9.1%)	0
Neutropenia	6 (18.2%)	1 (3%)
Thrombocytopenia	4 (12.1%)	0
Hypertension	3 (9.1%)	0
Azotemia	7 (21.2%)	1 (3%)
Bleeding	0	0
Thrombosis	0	0
Infection	3 (9.1%)	0
Nausea and vomiting	4 (12.1%)	0
Anorexia	3 (9.1%)	0
Peripheral neuropathy	18 (54.5%)	2 (6.1%)
Diarrhea	5 (15.2%)	0
Oral mucositis	6 (18.2%)	1 (3%)
Allergic reaction	1 (3%)	0

## 4. Discussion

In this study, we investigated whether normalization of posttreatment SCC Ag could be used as a prognostic marker for patients with advanced cervical cancer who were treated with bevacizumab and TP. We found that first-line chemotherapy with bevacizumab and TP was effective in patients with advanced cervical cancer in South Korea.

These findings are consistent with those of previous studies,^[15–17]^ although the survival outcomes in this study cohort were markedly longer. Tumor neovascularization plays a key role in the aggressive progression of cervical cancer, and the overexpression of oncogenic HPV subtypes enhances VEGF expression. The prevalence of high-risk HPV infection is higher in the Asian region compared with that in Europe and the Northern American regions, suggesting that anti-VEGF agents may have a more pronounced effect among Asian patients with cervical cancer. Moreover, most patients in this study exhibited good tolerance to the chemotherapy regimen, likely because of the effective supportive care provided by experienced medical oncologists.

Identification of prognostic or predictive factors for patients most likely to benefit from bevacizumab remains a considerable challenge. Interestingly, normalization of SCC antigen levels during chemotherapy was the only factor significantly associated with prolonged OS in patients with advanced cervical cancer. This finding is consistent with previous studies that have identified SCC Ag as a prognostic marker in patients with cervical cancer.^[15,16]^ Elevated pretreatment SCC Ag levels correlated with advanced disease characteristics, including FIGO stage, tumor size, depth of stromal invasion, lymphovascular space involvement, parametrial spread, lymphatic involvement, and poor survival outcomes.^[17,18]^ Elevated posttreatment SCC Ag levels correlated with poor prognosis, serving as a valuable marker for identifying patients at high risk of tumor persistence or early recurrence.^[15,19]^ To the best of our knowledge, this study is the first to show that normalization of SCC antigen levels during bevacizumab and TP chemotherapy is associated with improved OS in patients with advanced cervical cancer.

Despite these promising results, this study had several limitations. First, the retrospective study design may have introduced a selection bias and inherent challenges related to missing data. Second, this study includes only 33 patients, which significantly limits statistical power. Third, the absence of a centralized radiology review, variability in imaging modalities, and differences in scan intervals are additional potential sources of bias. Fourth, the prognostic role of SCC Ag normalization is hypothesis-generating, and prospective validation in larger, multi-center cohorts is necessary before SCC Ag can be used as a routine biomarker in clinical decision-making. However, these limitations reflect real-world clinical practice and the heterogeneous nature of the oncological care. Finally, the absence of a comparator arm precludes definitive conclusions regarding whether bevacizumab–TP combination therapy is superior or at least equivalent to other therapeutic options for advanced cervical cancer.

In conclusion, this study highlights that first-line chemotherapy with bevacizumab and TP is both effective and well-tolerated among Korean patients with advanced cervical cancer. Furthermore, the normalization of posttreatment SCC antigen levels may serve as a potential prognostic marker for improved OS. Notably, the response rates and OS observed in this cohort were superior to those reported in Western populations, suggesting that combination therapy could be particularly effective for Asian patients.

## Author contributions

**Conceptualization:** Haa-Na Song.

**Formal analysis:** In Bong Ha.

**Investigation:** Jae Min Cho.

**Resources:** Bong Hoi Choi.

**Supervision:** Jeong Kyu Shin.

**Validation:** Jeong Hee Lee.

**Writing – original draft:** Haa-Na Song.

**Writing – review & editing:** Haa-Na Song, In Bong Ha, Bae Kwon Jeong, Jae Min Cho, Bong Hoi Choi, Jeong Hee Lee, Jeong Kyu Shin.
